# Comparison of perioperative and functional outcomes of single-incision versus standard multi-incision robot-assisted laparoscopic radical prostatectomy: a prospective, controlled, nonrandomized trial

**DOI:** 10.1007/s11701-024-01962-2

**Published:** 2024-05-03

**Authors:** Shida Fan, Zhengjun Chen, Fang Zhou, Qian Lv, Dong Wang, Shangqing Ren, Xuemei Tian

**Affiliations:** 1 Robotic Minimally Invasive Surgery Center, Sichuan Provincial People’s Hospital, School of Medicine, University of Electronic Science and Technology of China, Chengdu, China; 2 Centre for Surgical Anaesthesia, Sichuan Provincial People’s Hospital, School of Medicine, University of Electronic Science and Technology of China, Chengdu, China

**Keywords:** Robot-assisted radical prostatectomy, Improved technology, Prostate cancer, Extraperitoneal implementation

## Abstract

To compare perioperative and functional outcomes between improved (port-free) single-site robot-assisted laparoscopic radical prostatectomy (pf-ssRARP) and standard multi-port robot-assisted radical prostatectomy (MPRARP). A total of 372 consecutive patients underwent RARAP using the da Vinci Si^®^ robotic surgical system. Group I (*n* = 210) included patients undergoing pf-ssRARP and Group II (*n* = 162) included patients undergoing MPRARP. Demographics and perioperative data including postoperative recovery outcomes were recorded and compared between the two groups. Overall mean operative time was significantly shorter with the pf-ssRARP compared to the MPRARP (*p* < 0.05). The length of hospitalization after the pf-ssRARP was shorter (*p* < 0.05). In Group I, the positive surgical margin rate was 15.2%; while in Group II, the positive margin rate was 33.3% (*p* < 0.05). The rate of instant urinary continence was significantly higher in Group I than in Group II (*p* < 0.05). The percentage of urinary continence was higher in the pf-ssRARP than in the MPRARP, at 6 months post-surgery (*p* < 0.05) and 9 months post-surgery (*p* < 0.05). There was no significant difference in the proportion of erectile function in the pf-ssRARP and MPRARP groups at the time of reaching the endpoint of this study (*p* > 0.05). The two groups were comparable in terms of total hospitalization costs (*p* < 0.05). The improved (port-free) single-site robot-assisted laparoscopic radical prostatectomy is a practical and easy technique to implement in clinical practice. Extraperitoneal implementation of the modified technique requires only a small incision, no special PORT, no additional auxiliary foramen creation, increased postoperative aesthetics and reduced hospitalization costs, and a high percentage of early postoperative urinary control recovery.

## Introduction

Prostate cancer (PCa) is the second most common cancer among men globally [1]. Between 2000 and 2018, the age-standardized mortality rate (ASMR) for cancer declined by about 1.2 percent per year among Chinese men, but there were increases in the incidence of prostate, colorectal, and pancreatic cancers [2]. Radical prostatectomy (RP) was the most common treatment for clinically localized intermediate- and high-risk prostate cancer [3, 4]. RP was traditionally performed via an open approach, but laparoscopic RP (LRP) and robot-assisted RP prostatectomy (RARP) have gained significant popularity in many health care systems [5]. For further minimization of surgical invasiveness, RARP is currently the accepted standard of care for surgically managing patients with localized prostate cancer [6]. During RARP, the surgeon has a three-dimensional view of the operating field that should mimic open surgery better than the two-dimensional view with the laparoscopic technique, and RARP incorporates high-level resolution and enlarged images as well as excellent lighting conditions [7].

Over time, there has been an increasing emphasis on performing surgery through fewer incisions to optimize the anatomic approach, improve aesthetics, and potentially reduce postoperative pain and adhesions [8]. With further advancements in technology, the single-port robotic surgical system is applied in clinical practice [9, 10]. Unlike traditional robotic surgical systems, the single-port robotic surgical system requires only one skin incision and a dedicated port to complete the surgical procedure.

To circumvent the limitations of special port and reduce patient hospitalization costs, we have made improvements on the single-port robotic surgical system for radical prostatectomy without the need for special ports. During the introduction of novel technology, one of the most important questions to assess is the safety and clinical efficacy of the device in question. Here, we offer the first prospective, controlled, nonrandomized study that demonstrates the technical safety and feasibility of the improved (port-free) single-site robot-assisted laparoscopic radical prostatectomy (pf-ssRARP). we sought to describe our experience, surgical technique, and outcomes regarding pf-ssRARP within a consecutive cohort of 210 patients.

To the best of our knowledge, this is the first prospective study comparing improved (port-free) single-site robot-assisted laparoscopic radical prostatectomy and standard multi-port robot-assisted radical prostatectomy. It is worth mentioning that we build preoperative three-dimensional reconstruction for precise preoperative planning based on computed tomography (CT) or magnetic resonance imaging (MRI) data.

## Patients and methods

Following approval from the ethics committee of Sichuan Provincial People’s Hospital, University of Electronic Science and Technology of China (2020010), data were prospectively collected by investigators from the Robotic Minimally Invasive Surgery Center, Sichuan Provincial People’s Hospital (Chengdu, China), from January 2021 to June 2023. Inclusion criteria: tumor stage cT1, cT2, or cT3a; imaging to exclude lymph node metastasis, organ metastasis and bone metastasis; cardiorespiratory fitness to tolerate general anesthesia surgery. A total of 372 consecutive patients underwent robot-assisted laparoscopic radical prostatectomy using the da Vinci Si® robotic surgical system (Intuitive Surgical Inc, Sunnyvale, CA, USA). Informed consent was obtained from all patients for being included in the study. Group I (*n* = 210) included patients undergoing the improved (port-free) single-site robot-assisted laparoscopic radical prostatectomy and Group II (*n* = 162) included patients who underwent standard multi-port robot-assisted radical prostatectomy. Patients were selected following physical examination, prostate specific antigen (PSA) screening, transrectal ultrasound-guided 12-core prostate biopsy, and cross-sectional imaging as indicated. All patients were diagnosed with organ-confined prostate cancer. Written informed consent was obtained from all patients after discussion of the use of the new surgical platform and technique. All surgeries were performed by a single surgeon with previous experience over 3000 cases of robotic surgery including prostate, kidney and bladder procedures and experience in robotic and laparoscopic single port surgery. Data were prospectively collected and include perioperative information regarding patient age, extra-prostatic extension., body mass index (BMI), American Society of Anesthesia (ASA) score, pre-operative cancer risk stratification, previous abdominal surgeries, International Index of Erectile Function (IIEF-5), International Prostatic Symptoms Score (IPSS),operative time, estimated blood loss, conversion rate, length of hospital stay, and complications. Extra-prostatic extension and prostate volume were measured by three-dimensional reconstruction technique. Time of crucial steps of the procedure was recorded separately: incision time and console time. Conversion was defined as the use of any additional port, change to open approach. Complications were assessed intraoperatively or during hospital stay using the Clavien–Dindo classification [11], and classified as a major complication (Clavien ≥ 3) and a minor complication (Clavien ≤ 2).Final pathological stage, surgical margins and prostate size were reported after histopathological evaluation of all prostatectomy specimens.

Regular follow-up of postoperative patients at 1 month, 3 months, 6 months, 9 months, 12 months, 18 months, 24 months, and 30 months, and all patients completed at least 6 months of follow-up. Sexual function were assessed utilizing the International Index of Erectile Function Questionnaire (IIEF) questionnaires [12]. Urinary continence was defined as using 0 pad or 1 pad for protection in 24 h. Biochemical recurrence was diagnosed postoperatively, on the first occasion after prostatectomy with a serum PSA was 0–2 ng/mL or greater and was confirmed by pathology (for each of the timepoints; 1 month, 3 months, 6 months, 9 months, 12 months, 18 months, 24 months, and 30 months). Postoperative patient imaging was discretionary with CT or MRI and radionuclide bone scans predominantly used, for evidence of metastases. We defined radiological progression as local or distant metastasis captured via imaging by MRI, bone scan, CT, or PET.

### Statistical analysis

Normal distribution of data was examined with one sample Kolmogorov–Smirnov test. Univariate analysis was performed using parametric (student’s *t*-test) and non-parametric (Mann–Whitney *U* test) testing for continuous variables and chi squared for categorical variables, as appropriate. *p*-values < 0.05 were considered statistically significant.

### Surgical technique

**Figure1:**
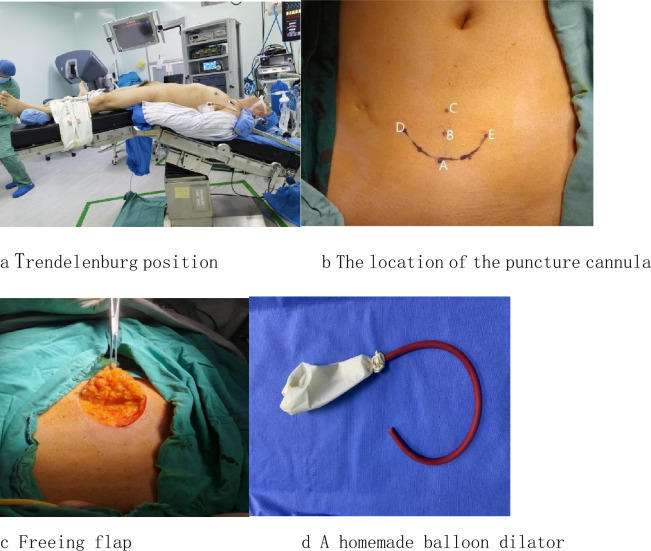

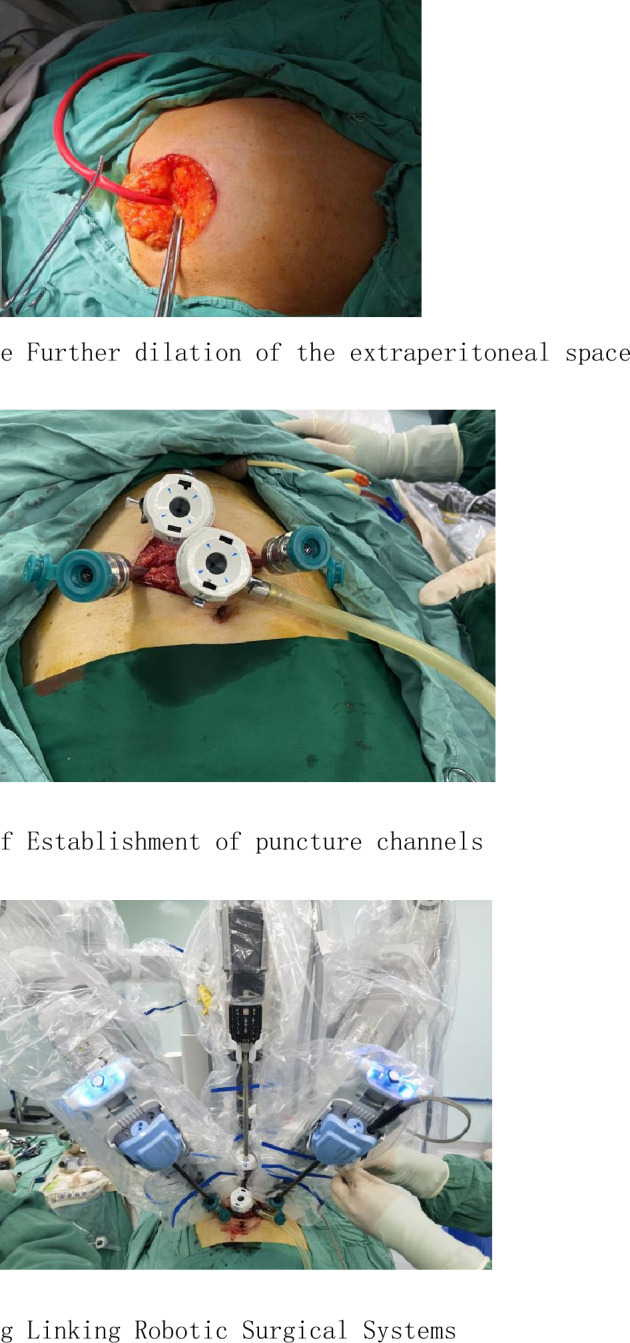
Establishing extraperitoneal space. **a** Trendelenburg position. **b** The location of the puncture cannula. **c** Freeing flap. **d** A homemade balloon dilator. **e** Further dilation of the extraperitoneal space. **f** Establishment of puncture channels. **g** Linking robotic surgical systems

Both approaches patients were placed under Trendelenburg position with both arms tucked (Fig. 1a). Preoperative preparation includes subcutaneous heparin and antibiotics prophylaxis (e.g., a cephalosporin).

### Approach

#### Improved (port-free) single-site robot-assisted laparoscopic radical prostatectomy(Group I)

##### Establishing extraperitoneal space

After emptying the bladder with an indwelling catheter, using the index and middle fingers to search for the upper edge of the pubic symphysis and marking a point. A single 5 cm arc incision is made as following: the lowest point of the arc incision was taken from the anterior midline to 5 cm on the pubic symphysis, and 2.5 cm on both sides of the 7–8 cm midline from the pubic symphysis was used as the arc incision at both ends (Fig. 1b);dissection of the abdominal planes are carefully done until reaching the anterior.rectus fascia,the space between the subcutaneous tissue and the rectus abdominis was fully freed, and the skin fap was turned to the cephalic side and pulled (Fig. 1c). Cutting the anterior sheath of the rectus abdominis muscle longitudinally at a distance of about 3 cm from the lowest point of the curved incision on the midline of the abdome. Blunt dissection is done using the index finger thru the facial incision to create an initial space under the anterior rectus fascia in the preperitoneal space. Next, a homemade balloon dilator (Fig. 1d) is introduced through the facia incision toward the pubic bone and the tip positioned in the pre-pubic bone area. Adequate working space is then developed by inflating the balloon with 900 cc of air for 10 s (Fig. 1e). A 12 mm trocar was inserted through the incision of the anterior sheath of the rectus abdominis,and a retraction stitch is taken at the inferior edge of the incision in order to maintain airtightness. After the extraperitoneal space is insufflated, the 30 degrees lens is inserted for observation and the remaining three puncture channels are established under visualization. The lower edge of the arc incision was pulled, the 12 mm trocar was placed at 3–4 cm above the pubic symphysis under direct vision, both ends of the arc incision were pulled, and two robotic metal puncture kits were placed at 3–4 cm on both sides of the midline (Fig. 1f). The da Vinci Si robot-assisted laparoscopic surgery system is then introduced into the surgical field and docked to the trocars.the robotic instruments are advanced into the extraperitoneal space under visualization (Fig. 1g). The procedure then proceeds nearly identically to the standard anterior approach robotic radical prostatectomy.

#### Standard multi-port robot-assisted radical prostatectomy(Group II)

##### Establishing transperitoneal space

A single-use 12 mm trocar was placed one centimeter above the umbilicus, and carbon dioxide gas was injected into the abdominal cavity to maintain a pneumoperitoneum pressure of 15 mmHg. Under direct vision, an 8 mm robotic metal cannula was placed 1.5–2.0 cm below the level of the paraumbilical region of the right lef lateral rectus abdominis and 8–10 cm from the lens hole. Te No. 1 and No. 2 mechanical arms were placed. Te 8 mm casing was placed 1.5–2.0 cm above the No. 2 manipulator, and the No. 3 manipulator was placed on the lef axillary front line of the No. 2 arm 8–10 cm. Te 12 mm cannula was placed as the helper hole 4 cm on the right side of the umbilical plane lens hole and 4 cm on the outside of the right mechanical arm, and the operation was performed using a transperitoneal approach. The procedure then proceeds nearly identically to the standard anterior approach robotic radical prostatectomy.

### Troubleshooting topic and limitations

We conducted a prospective, controlled, non-randomized trial, which introduced a bias in the selection of cases, and we will randomize all subjects in subsequent studies as the number of cases continues to increase, so that the results will be more accurate. In our study, we only performed three-dimensional reconstruction in Group I, which may affect the comparison of some evaluation indexes; to continue to integrate three-dimensional reconstruction with robotic surgical techniques, preoperative three-dimensional evaluation of all cases will be performed in our follow-up multicenter study. We will conduct a multicenter, prospective, controlled, randomized trial to evaluate the new technique, with surgical access through the extraperitoneal route, which avoids bias due to differences in surgical access.

## Results

During the study period, 372 patients underwent robot-assisted laparoscopic radical prostatectomy, 210 performed the improved (port-free) single-site robot-assisted laparoscopic radical prostatectomy and 162 performed the standard multi-port robot-assisted radical prostatectomy.

### Pre-operative data

**Figure2:**
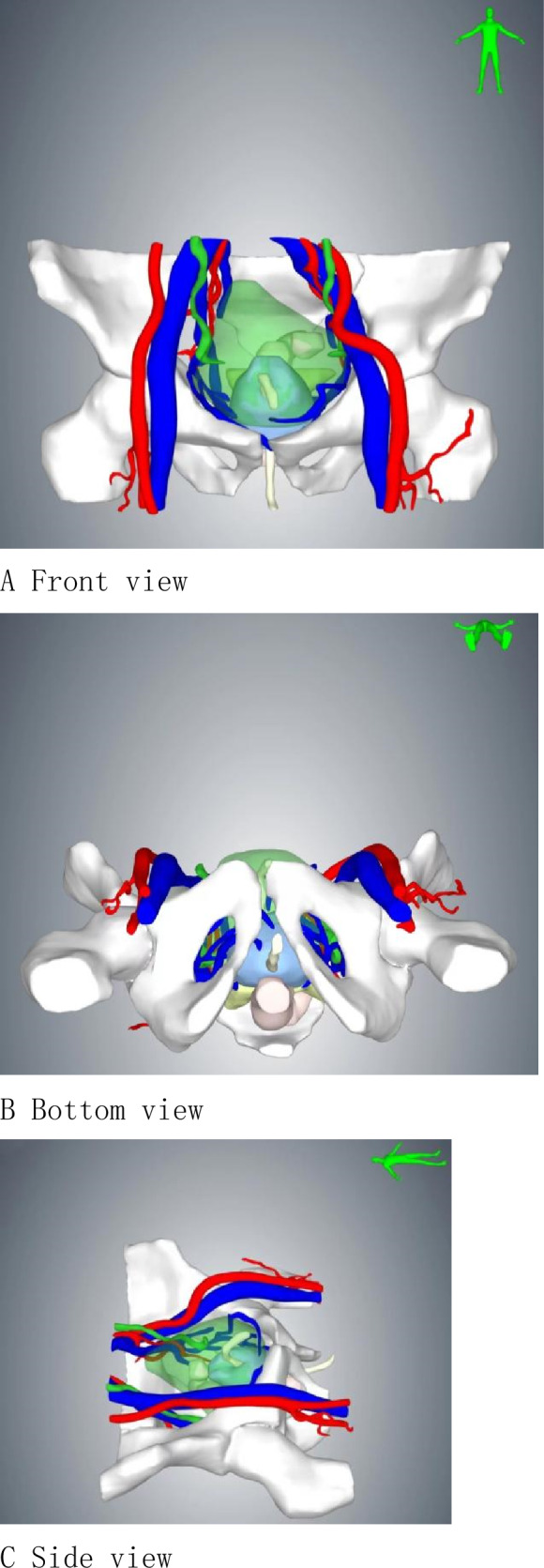

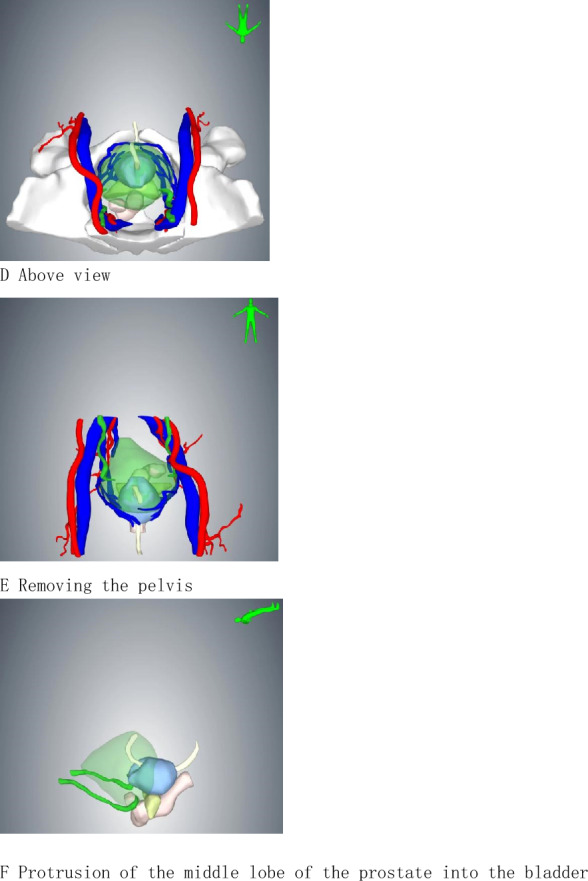
Three-dimensional reconstruction. **A** Front view. **B** Bottom view. **C** Side view. **D** Above view. **E** Removing the pelvis. **F** Protrusion of the middle lobe of the prostate into the bladder

No significant differences were observed in mean age (*p* = 0.99), body mass index (*p* = 0.91), American Society of Anesthesiologists score (*p* = 0.69), preoperative prostate specific antigen (*p* = 0.51), or National Comprehensive Cancer Network risk group (*p* = 0.10), extraprostatic extension (*p* = 0.45)between the groups. Of note, 49 patients in this cohort had undergone intra-abdominal surgeries, such as cholecystectomy, appendectomy, radical rectal cancer, colectomy, and hernia repair. Summary of patient demographics and pre-operative data is shown in Table 1.The three-dimensional reconstruction view is shown in Fig. 2.

**Table 1:** Demographics and pre-operative data

Characteristic	pf-ssRARP (*n* = 210)	MPRARP (*n* = 162)	*p *value
Age, year (mean, range)	64 (55–71)	66(53–72)	0.99
Preoperative PSA level, ng/mL (mean, range)	7.9(5.5–10.6)	8.3(5.3–12.1)	0.51
American Society of Anesthesiologists (ASA) Score (mean, range)	1.6(1–3)	1.7(1–3)	0.69
Prostate volume^a^, cm^3^ (mean, range)	67(55–78)	63(53–80)	0.75
Extra-prostatic extension^a^, *n*(%)	106(50.4)	89(54.8)	0.377
BMI (kg/m^2^) (mean, range)	26(23–29)	27(22–31)	0.91
Previous abdominal surgery, *n*(%)	23(11.0)	26(16.0)	0.12
Preoperative continence, *n*(%)	208(99.0)	160(98.7)	0.88
IPSS, *n*(%)			0.84
Mild 0–7	150(71.4)	109(67.3)	
Moderate 8–19	43(20.5)	42(26.0)	
Severe 20–35	17(8.1)	11(6.7)	
Preoperative biopsy Gleason score, *n*(%)			0.76
< 7	89(42.3)	63(38.9)	
7	60(28.6)	50(30.8)	
> 7	61(29.1)	49(30.3)	
Preoperative clinical tumor stage, *n*(%)			0.65
cT1	47(22.3)	31(19.2)	
cT2	130(62.0)	96(59.2)	
cT3a	33(15.7)	35(21.6)	
IIEF^b^ score, *n*(%)			0.73
> 21	83 (39.5)	54 (33.3)	
< 21	127 (60.5)	108 (66.7)	

*IPSS* international prostatic symptoms score, *IIEF* International Index of Erectile Function

^a^Measurement by three-dimensional reconstruction technique

^b^IIEF Questionnaire modified version with five questions, six answer categories, 0–5 points per question, score ≤ 16 = erectile dysfunction, score ≤ 21 = some erectile function, score > 21 = no erectile dysfunction

### Intraoperative data and postoperative data

Intraoperative and postoperative data are presented in Table 2. Overall mean operative time was significantly shorter with the pf-ssRARP compared to the MPRARP (83.5 ± 12.6 versus 106.2 ± 16.8 min;* p* < 0.05). The time of key steps of the surgery was recorded separately, such as the time to establish the surgical space, the time at the console, and the time to close the surgical incision. The console time for patients who underwent improved technology is shorter than those who underwent traditional surgery (46.5 ± 5.7 versus 71.4 ± 12.6 min; *p* < 0.05). The comparison of estimated blood loss between the two groups was not statistically significant, and there were no patients who received blood transfusions due to excessive bleeding. The length of hospitalization after the improved technology was shorter, with a median (IQR) of 5.6 days (4–7 days), compared to 8.9 days (5–13 days) in the control group (*p* < 0.05). Patients undergoing pf-ssRARP treatment had a significantly shorter indwelling catheter duration compared to those receiving MPRAR treatment (4 vs 7 days, *p* < 0.05). On pathology, in Group I patients, the positive surgical margin rate was 15.2%, while in Group I patients, the positive margin rate was 33.3% (*p* < 0.05). The positive surgical margin could be present at the apex or base of the prostate in both groups, but most were located at the base of the prostate. The extra-prostatic extension of patients in Group I and Group II are 50.4 and 54.8%, respectively (*p* = 0.377). The postoperative complications rates for Group I and Group II were 10.0 and 11.1% (*p* = 0.423), respectively, which were not statistically significant. In neither of the two surgical procedures did we routinely perform pelvic lymph node dissection. The total hospitalization costs (RMB) for pf-ssRARP and MPRAR were 70,762.3 ± 5745 and 79,524.7 ± 6372, respectively, with statistically significant differences. Recovery time of bowel function after pf-ssRARP was significantly faster than MPRAR (23.3 ± 4.9 versus 37.8 ± 5.3 h; *p* < 0.05).

**Table 2:** Intraoperative, postoperative, and follow-up data

Characteristic	pf-ssRARP (*n* = 210)	MPRARP (*n* = 162)	*p *value
Overall operative time, min (mean ± SD)	83.5 ± 12.6	106.2 ± 16.8	< 0.05
Console time	46.5 ± 5.7	71.4 ± 12.6	< 0.05
Estimated blood loss, mL (mean ± SD)	165 ± 89	173 ± 94	0.76
Length of hospital stay (IQR)	5.6 (4–7)	8.9 (5–13)	< 0.05
Catheter removal, days (mean ± SD)	4 ± 1.3	7 ± 2.8	< 0.05
Positive surgical margins, *n*(%)	32(15.2)	54(33.3)	< 0.05
Conversion to traditional RARP, *n*(%)	0(0)	0(0)	
Prostate specimen weight, gr (mean ± SD)	57.3 ± 23.3	62.1 ± 21.6	0.77
Duration of anal defecation, hours (mean ± SD)	23.3 ± 4.9	37.8 ± 5.3	< 0.05
Total costs, ¥ (mean ± SD)	70,762.3 ± 5745	79,524.7 ± 6372	< 0.05
Pathologic TNM stage, *n*(%)			0.72
pT2	142(67.6)	111(68.5)	
pT3	62(29.5)	48(29.6)	
pT4	6(2.9)	3(1.9)	
Complications (Clavien–Dindo), (Grade, *n*)	21/210(10.0%)	18/162(11.1%)	0.423
I	Anastomosis leakage(7)	Anastomosis leakage(4)	
II	Urinary tract infection(3)	Urinary tract infection(2)	
	Postoperative anemia(1)	Pulmonary embolism(1)	
		Postoperative anemia(1)	
III	Pelvic abscess(2)	Pelvic abscess(3)	
	Pelvic hematoma(3)	Pelvic hematoma(4)	
	Inguinal hernia(5)	Umbilical hernia(3)	
Follow-up, months (mean ± SD)	20 ± 6.5	19 ± 5.9	0.83
Continence rate^a^, *n*(%)			
Instant urinary continence^b^	103(49)	44(27)	< 0.05
6-month continence	143(68)	73(45)	< 0.05
9-month continence	185(88)	112(69)	< 0.05
12-month continence	192(91)	143(88)	0.373
Erectile function, IIEF^c^ score, *n*(%)			
IIEF > 16	41(19.2)	30(18.7)	0.887
Biochemical recurrence, *n*(%)	5(2.3)	8(4.9)	0.445

^a^Urinary continence was defined as using 0 pad or 1 pad for protection in 24 h

^b^Instant urinary continence was defined as the ability to complete the act of urination successfully when the catheter was removed for the first time

^c^IIEF questionnaire modified version with five questions, six answer categories, 0–5 points per question, score ≤ 16 = erectile dysfunction, score ≤ 21 = some erectile function, score > 21 = no erectile dysfunction

### Follow-up outcomes

Instant urinary continence was defined as the ability to complete the act of urination successfully when the catheter was removed for the first time. In our study, the rate of instant urinary continence was significantly higher in Group I than in Group II (49% versus 27%; *p* < 0.05). The percentage of urinary continence was higher in the improved (port-free) single-site robot-assisted laparoscopic radical prostatectomy group than in the standard multi-port robot-assisted radical prostatectomy group, at 6 months post-surgery (68% versus 45%; *p* < 0.05) and 9 months post-surgery (88% versus 69%;* p* < 0.05)., but there was no significant difference in the urinary continence rates between the two groups after 12 months of follow-up (91% versus 88%; *p* = 0.373), until the end of the study. Throughout the entire follow-up period, sexual function scores (measured by the IIEF) did not differ significantly between the improved (port-free) single-site robot-assisted laparoscopic radical prostatectomy group and standard multi-port robot-assisted radical prostatectomy group. Disease progression, including biochemical recurrence or metastasis on imaging, occurred in five cases in the modified surgery group and eight cases in the conventional surgery group at 30-month follow-up (2.38% versus 4.93%; *p* = 0.467).

## Discussion

In this article, we present an extraperitoneal improved (port-free) single-site robot-assisted laparoscopic radical prostatectomy for prostate cancer using the da Vinci Si robotic surgical system (Intuitive Surgical Inc, Sunnyvale, CA, USA) and compare it with the transperitoneal standard multi-port Robot-assisted Radical Prostatectomy using the same robotic surgical platform. Our initial aim in improving the technology was to get rid of the special PORT limitations and to reduce the cost of hospitalization for the patients.

Radical prostatectomy (RP) remains the most effective method for treating localized prostate cancer, and in 1991 Schuessler were the first to report laparoscopic radical prostatectomy(LRP) [13]. With the continuous development of artificial intelligence technology and medical robotics, Binder first reported conducting robot-assisted laparoscopic radical prostatectomy (RARP) in 2001 [14]. To further reduce surgical trauma and fully utilize robotic surgical assistance systems to minimize perioperative complications and speed up postoperative patient recovery, Kaouk first reported the single-port robotic-assisted laparoscopic radical prostatectomy (spRARP) in 2009 [15].Many medical centers at home and abroad have now established RARP as the gold standard for treating localized prostate cancer, while spRARP has been gradually carried out [16, 17].The shift from traditional “multi-wound” laparoscopic surgery to “single-wound” laparo-endoscopic single-site surgery (LESS) has been driven by the trend towards a minimally invasive approach [18], but single-site robotic techniques often rely on special disposable multichannel laparoscopic surgical access devices (referred to as specialized PORT) [19]. When we carried out ssRARP in the previous period, matching problems, instrument interference, channel leakage, reverse motion, and high cost limited the promotion and popularity of this technique. To solve the many limitations of the dedicated PORT, we have innovated and improved (port-free) single-site robot-assisted laparoscopic radical prostatectomy. We see the main advantages of not relying on PORT as follows: (1) Surgical space and operation are affected to a certain extent by the possible displacement and instability of the airtightness of the multi-channel single-port laparoscopic surgical puncture device during surgery; (2) Due to the multi-channel single-port laparoscopic surgical puncture device, the puncture point of the instrument is higher than the surface of the body, and there is a “projection effect”, which shortens the distance of the instrument arm in the body cavity, makes it easy to fight, and restricts the operation range; (3) Multi-channel single-port laparoscopic surgical puncture devices are more expensive, increasing patient costs; (4) Some medical institutions are constrained by specialized puncture devices and have cumbersome application procedures and long lead times, making it difficult to carry out single-hole techniques.

In the prospective controlled nonrandomized trial, we used two surgical approaches, transabdominal and extraperitoneal, to perform radical prostatectomy. Improved technology for surgical operation via the extraperitoneal route, the entire surgical process does not go through the abdominal cavity, less interference with abdominal organs, reducing the possibility of damage to abdominal organs, especially the intestines, and faster recovery of the patient’s intestinal function after surgery. In our study, the time to bowel evacuation was significantly shorter in patients after pf-ssRARP than in patients after MPRARP. For postoperative patients with bowel evacuation, we advise them to drink water appropriately and then gradually transition from eating to a liquid diet or normal diet, while reducing the patients’ intravenous nutritional support. If the postoperative patient’s bowel recovery time is fast, then early release from parenteral nutritional support can reduce hospitalization costs. It is worth noting that, in view of the special nature of the surgical site, when the patient’s intestinal function recovers, we will allow the patient to take some medication to help defecation to prevent constipation in the postoperative period. For patients with a history of abdominal surgery, pf-ssRARP can proceed without considering abdominal adhesions, whereas MPRARP needs to take abdominal adhesions into account. If adhesions are present, adhesiolysis should be performed first, which will increase operative time as well as the risk of intestinal injuries. The modified technique incorporates multiple abdominal wall puncture channels into a small incision in the lower abdomen, resulting in a less invasive and more aesthetically pleasing procedure. This approach minimizes scarring and reduces recovery time, offering a more favorable outcome for patients. The enhanced precision of the procedure also contributes to decreased post-operative discomfort and allows for a quicker return to daily activities. Modified technology requires only the lens arm, arm 1, arm 2, and an auxiliary hole, whereas conventional technology requires arm 3 and an additional auxiliary hole. During the implementation of the improved technique, the assistant only needs to use the suction device through a single auxiliary hole to maintain a clear surgical field of view and to assist the primary surgeon in fully exposing the surgical field during critical surgical steps. In contrast, traditional techniques require the assistant to alternately use the suction device and atraumatic forceps during the surgical procedure. This higher level of coordination between the assistant and the primary surgeon in the improved technique may be one of the reasons for the shorter duration of the surgery compared to traditional techniques. It keeps the surgical team focused, and the streamlined process allows for more efficient collaboration. The improved technique truly showcases how advancements in surgical methods can positively impact operational procedures and patient outcomes.

Overall mean operative time was significantly shorter with the pf-ssRARP (83.5 min) compared to the MPRARP (106.2 min) (*p* < 0.05). In this study, we recorded the time to establish the surgical space, the time at the console, and the time to close the surgical incision, respectively. The console time for patients who underwent pf-ssRARP (46.5 min) is shorter than those who underwent MPRARP (71.4 min) (*p* < 0.05). With the development of digital technology, three-dimensional reconstruction technique based on enhanced CT and MRI data can vividly reflect human anatomical structures, aiding surgeons in precisely formulating preoperative planning [20, 21]. In our study, for patients undergoing pf-ssRARP, we performed preoperative three-dimensional reconstruction, which allowed us to visualize preoperatively the anatomical relationship of the prostate to the surrounding structures, in particular the extent to which the middle lobe of the prostate protrudes into the bladder, which in turn allowed us to personalize the procedure. The purpose of preoperative planning using three-dimensional reconstruction is to be able to simulate the surgical steps prior to the surgery, so that difficulties that may be encountered during the surgery can be anticipated as well as to save surgical time. The improved technology through the extraperitoneal route for surgical operations to reduce the interference with the abdominal cavity organs, the patient’s intestinal tract and other organs after surgery to recover quickly, and modified technology short operating time, less trauma conducive to the postoperative patients to get out of bed as soon as possible, so the hospitalization time of the modified technology is shorter.

The radical prostatectomy has undergone a lengthy development process from the initial open procedure to the current robotic-assisted laparoscopic technique, all aimed at advancing the surgical approach toward a more minimally invasive direction. In recent years, the use of robotic-assisted laparoscopic technology in radical prostatectomy has become increasingly widespread. Leveraging the advantages of the da Vinci surgical robot system, we have made many improvements to the traditional procedure. Initially, our main aim was to achieve better perioperative outcomes, such as shorter operative times, less intraoperative bleeding, lower rates of postoperative complications, and shorter postoperative patient hospital stays, and eventually our aim was gradually achieved. But only having this as the goal is far from enough, now we need to consider more about the long-term follow-up results of the patients after surgery, obtaining a good quality of life after surgery is even more important. The extent and duration of recovery of urinary continence after radical prostatectomy is now a greater concern for physicians as well as for patients. In China, patients are more concerned about the recovery of urinary continence compared to preservation of sexual function. Although the problem of long-term urinary incontinence following robotic-assisted radical prostatectomy (RARP) for prostate cancer can be considered almost solved, with urinary continence rates as high as 97% at 12 months postoperatively, immediate and early incontinence continues to have a negative impact on patients’ quality of life [22]. In our study, we recorded the recovery of immediate urinary continence (IC), early urinary continence (EC) (6 and 9 months postoperatively), and long-term urinary continence (LC) (after 12 months postoperatively) in two groups of patients after surgery. The study found that the rate of IC was significantly higher in pf-ssRARP than in MPRARP (49% versus 27%; *p* < 0.05), and the percentage of EC was higher in pf-ssRARP than in MPRARP, at 6 months post-surgery (68% versus 45%; *p* < 0.05) and 9 months post-surgery (88% versus 69%;* p* < 0.05), but there was no significant difference in the LC rates between the two groups after 12 months of follow-up (91% versus 88%; *p* = 0.373). After undergoing radical prostatectomy, the exact reasons for postoperative urinary incontinence are not clear. However, the following factors can be considered as predictors of postoperative urinary incontinence: shorter membranous urethral length, older age, longer surgical duration, higher body mass index, lower preoperative serum albumin levels, history of transurethral prostate resection, wider bladder neck opening during surgery, preoperative urinary incontinence status, surgical technique, surgeon expertise, and postoperative factors [23–25]. To improve the patient's ability to recover early urinary continence (EC), surgical modifications have been made based on three means: (1) preserving as much anatomical integrity as possible, (2) reconstructing anatomical structures related to urinary continence, and (3) strengthening anatomical structures related to continence [26].In the pf-ssRARP group, we created an extraperitoneal space for radical prostatectomy, which allowed us to have direct access to the prostate and minimized unnecessary free manipulation, helping to preserve as much of the anatomy as possible in relation to urinary continence. Apart from this, the console time for patients who underwent improved technology is shorter than those who underwent traditional surgery (46.5 ± 5.7 versus 71.4 ± 12.6 min; *p* < 0.05).The above two aspects may explain the superiority of immediate urinary continence and early urinary continence in pf-ssRARP group over in MPRARP group. To eliminate the potential errors caused by individual surgical skills, all operations are carried out by a single surgeon with over 3000 cases of robotic surgery experience. But, there was no significant difference in the urinary continence rates between the two groups after 12 months of follow-up (91% versus 88%; *p* = 0.373). Many clinical trials have shown that there is no significant difference in long-term urinary continence rates after radical prostatectomy, which is similar to our research results [27, 28]. After RARP, the patient’s urinary continence function gradually recovers over time, reaching a stable state at 12 months postoperatively and all showing good urinary continence, but the specific mechanism is not yet clear [29]. The recovery of immediate and early continence is quite important for improving the postoperative quality of life for patients, and our improved techniques are helpful for the recovery of immediate and early continence.

As well as urinary incontinence, post-prostatectomy erectile dysfunction (ED) still pose a challenge that adversely affects the patient’s quality of life. The relevant literature reports that more than half of patients after radical prostatectomy will experience erectile dysfunction, and this proportion can reach 70% [30, 31]. There was no significant difference in the proportion of erectile function in the pf-ssRARP and MPRARP groups at the time of reaching the endpoint of this study, (43% versus 39%; *p* > 0.05). Research has shown that erectile function (EF) after radical prostatectomy has been closely associated with the extent of neural preservation and the use of an atraumatic and traction-free dissection [32, 33]. Tewari proposed a grading system for nerve preservation that involves identifying four different anatomical levels from which the degree of surgical nerve preservation varies [34]. To improve nerve-sparing, Pedraza et al. used saline-assisted fascial exposure(SAFE) technique to perform robotic-assisted radical prostatectomy, facilitating an atraumatic dissection of the neural hammock as well as the visualization of periprostatic nerves [35]. In their findings, the use of the SAFE technique led to better sexual health inventory for men(SHIM) scores at 6, 13, 26, and 52 weeks after RALP. In our study, the pf-ssRARP group performed radical prostatectomy via the extraperitoneal route, and compared with the MPRARP group via the transperitoneal route, we believe that the pf-ssRARP group had a higher use of non-invasive and traction-free dissection throughout the entire surgical procedure, which contributes to the recovery of postoperative sexual function; however, the statistical results showed that there was no significant difference in the proportion of sexual function recovery between the two groups. It can be seen that the recovery of sexual function after radical prostatectomy is affected by a number of factors.

We conducted a prospective, controlled, nonrandomized trial to compare perioperative data and follow-up data between the extraperitoneal improved (port-free) single-site robot-assisted laparoscopic radical prostatectomy and the transperitoneal standard multi-port robot-assisted radical prostatectomy. The results show that the modified technique is feasible, practical, and easy to replicate in the clinic. To make the results of the study more reliable, all of our surgeries were performed by the same lead surgeon with extensive experience in robotic surgical operations, and to prevent the initial lack of proficiency in the establishment of the extraperitoneal space, which would have some impact on the operation time, we have begun to implement a modified surgery in the early stage. However, there are still some shortcomings in our study: we did not randomly assign patients to the enrollment, and the patients chose the surgery voluntarily, but the attending surgeon may have some tendency to influence the final choice of the patients in the preoperative communication; on the other hand, we only performed three-dimensional reconstruction in patients who underwent the modified surgery, which may affect the comparison of some evaluation indexes; we have compared extraperitoneal versus transperitoneal route and this introduced a bias. We will follow up with a multicenter, prospective, controlled, randomized trial to further evaluate the improved technology.

## Conclusions

The pf-ssRARP has the advantages of shorter procedure time, faster postoperative recovery, less hospitalization cost, and faster recovery from early urinary control than the MPRARP, but there is no difference in sexual function recovery.

## Data Availability

No datasets were generated or analysed during the current study.
